# Dentinogenesis imperfecta type II in Swedish children and adolescents

**DOI:** 10.1186/s13023-018-0887-2

**Published:** 2018-08-22

**Authors:** K. Andersson, B. Malmgren, E. Åström, G. Dahllöf

**Affiliations:** 1 Department of Dental Medicine, Division of Orthodontics and Pediatric Dentistry, Karolinska Institutet, POB 4064, SE-141 04 Huddinge, Sweden; 2 Department of Women’s and Children’s Health, Karolinska Institutet, Stockholm, Sweden; 3 Pediatric Neurology, PO3, Astrid Lindgren Children’s Hospital, Karolinska University Hospital, Stockholm, Sweden

**Keywords:** Connective tissue, Dentin sialophosphoprotein, Dentin dysplasia, Genetic disorder, Osteogenesis imperfecta, Prevalence

## Abstract

**Background:**

Dentinogenesis imperfecta (DGI) is a heritable disorder of dentin. Genetic analyses have found two subgroups in this disorder: DGI type I, a syndromic form associated with osteogenesis imperfecta (OI), and DGI type II, a non-syndromic form. The differential diagnosis between types I and II is often challenging. Thus, the present cross-sectional study had two aims: to *(i)* investigate the prevalence and incidence of DGI type II among Swedish children and adolescents and *(ii)* search out undiagnosed cases of DGI type I by documenting the prevalence of clinical symptoms of OI in these individuals. We invited all public and private specialist pediatric dental clinics (*n* = 47) in 21 counties of Sweden to participate in the study. We then continuously followed up all reported cases during 2014−2017 in order to identify all children and adolescents presenting with DGI type II. Using a structured questionnaire and an examination protocol, pediatric dentists interviewed and examined patients regarding medical aspects such as bruising, prolonged bleeding, spraining, fractures, hearing impairment, and family history of osteoporosis and OI. Joint hypermobility and sclerae were assessed. The clinical oral examination, which included a radiographic examination when indicated, emphasized dental variables associated with OI.

**Results:**

The prevalence of DGI type II was estimated to be 0.0022% (95% CI, 0.0016–0.0029%) or 1 in 45,455 individuals. Dental agenesis occurred in 9% of our group. Other findings included tooth retention (17%), pulpal obliteration (100%), and generalized joint hypermobility (30%). Clinical and radiographic findings raised a suspicion of undiagnosed OI in one individual, a 2-year-old boy; he was later diagnosed with OI type IV.

**Conclusions:**

These results show a significantly lower prevalence of DGI type II than previously reported and point to the importance of excluding OI in children with DGI.

**Electronic supplementary material:**

The online version of this article (10.1186/s13023-018-0887-2) contains supplementary material, which is available to authorized users.

## Background

**Fig. 1:**
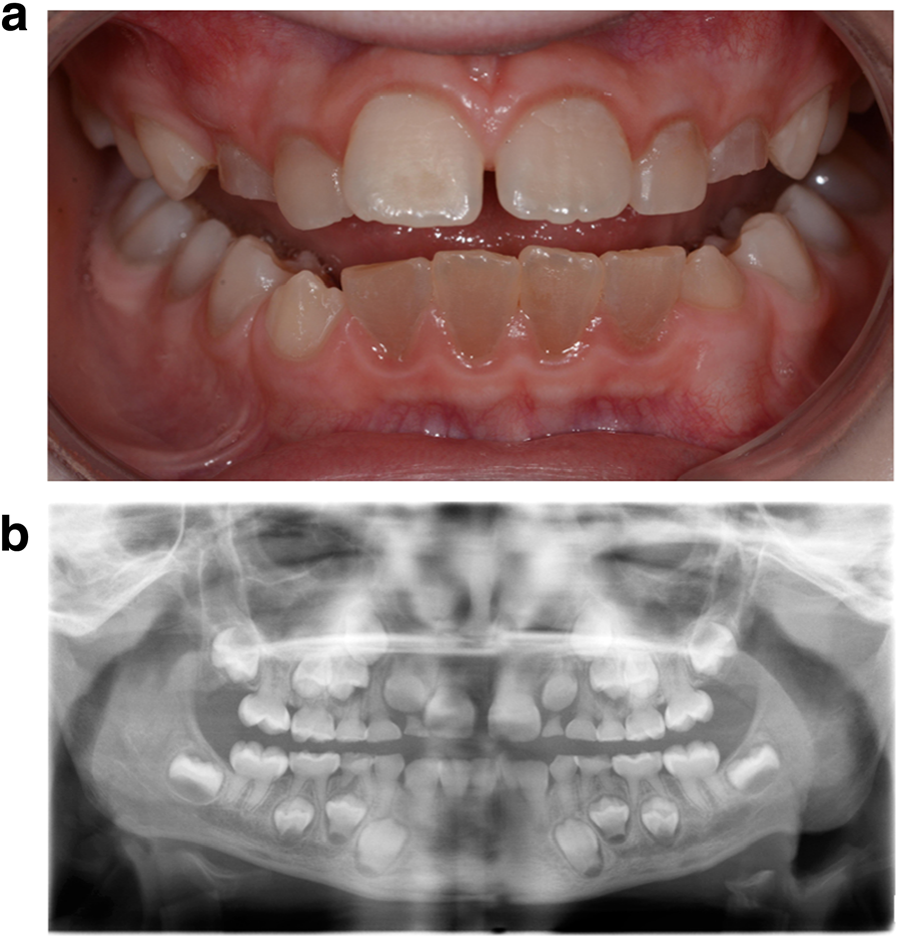
**a** A 10-year-old boy presenting with dentinogenesis imperfecta (DGI) type II in the mixed dentition. In the permanent dentition, the mandibular incisors are most severely affected. **b** Radiographic findings of DGI in the same boy at 6 years of age. Marked cervical constriction, varying degree of pulpal obliteration, and short roots are visible

Dentinogenesis imperfecta (DGI) is a heritable disorder of the dentin (the inner calcified tissue protecting the pulp, along with enamel and cementum). The mineral content and structure of the enamel are normal, but the enamel is easily fractured due to the underlying soft dysplastic dentin, which also causes the teeth to exhibit a grey-blue to brown discoloration (Fig. 1a). The dentin is prone to attrition, with the deciduous dentition often being more severely affected than the permanent. Radiographically, the teeth exhibit a morphology that is pathognomonic for the condition, with bulbous crowns, a marked cervical constriction, pulpal obliteration, and short roots (Fig. 1b). Based on genetic findings, two subgroups form this disorder: DGI type I (DGI-I), a syndromic form associated with osteogenesis imperfecta (OI); and DGI type II (DGI-II), the non-syndromic form, that is unassociated with OI or any other inherited disorder [1–4].

DGI-I (a collagenous disorder) is a common feature in osteogenesis imperfecta (OI). Depending on type of OI, reported prevalences of DGI-I in patients with OI range from 8 to 100%. Children with a more severe type of OI often present with a more severe dental phenotype [5–8].

The differential diagnosis between DGI types I and II is often challenging. DGI-II (a non-collagenous disorder) is an autosomal dominant disorder with a high degree of penetrance and expressivity. De novo mutations are rare. Mutations in the dentin sialophosphoprotein (*DSPP*) gene located on chromosome 4 (4q22.1) are the source of the condition. Clinical, radiographic, and histological findings in DGI types I and II are similar, although inter- and intra-individual variability of expression is greater in DGI-I. Due to similarities in expression of the two types, there is reason to believe that undiagnosed cases of mild OI may be present among individuals with DGI. To identify these children, evaluation of the variables associated with OI (bruising, prolonged bleeding, spraining, fractures, hearing impairment, joint hypermobility, blue sclerae, and family history of osteoporosis and OI) may be necessary.

Information regarding prevalence and incidence of DGI-II is scarce. In the United States, Witkop [9] found an incidence of 0.011−0.013% in a cohort of families in the state of Michigan in 1957. Due to the character of genetic isolate, there is reason to believe that the incidence is considerably lower in other populations compared to the individuals investigated by Witkop. Gupta et al. [10] found that DGI was the rarest developmental dental anomaly, presenting in 0.09% of the individuals in an Indian population. The prevalence of type II in a French population was estimated to be 0.057% [11] and 0.1% in Saudi children [12]. The Indian, French, and Saudi Arabian studies were single center cohorts and had retrospective designs.

The present study had two aims: to investigate the prevalence and incidence of DGI-II among children and adolescents throughout the Swedish population, and to search out undiagnosed cases of DGI-I by documenting the prevalence of clinical symptoms of OI in these individuals. We hypothesized that the prevalence of DGI-II is significantly lower than previously reported and that there are undiagnosed cases of OI in children and adolescents who were incorrectly diagnosed with DGI-II.

## Methods

We invited all public and private specialist pediatric dental clinics (*n* = 47) in the 21 counties of Sweden to participate in the study. At the 2013 Swedish Academy of Pediatric Dentistry meeting, we invited all pediatric dental specialists and residents in Sweden (*n* = 179) to participate in our planned study. All children and adolescents (0−19 years old) diagnosed with DGI-II, who were or previously had been in treatment, were asked for. During 2014−2017, we continuously followed up all reported cases in order to confirm all children and adolescents presenting with DGI-II.

### Clinical and radiographic examination

Using a structured questionnaire and an examination protocol with guidelines, pediatric dentists interviewed and examined all patients regarding medical aspects such as bruising, prolonged bleeding, spraining, fractures, hearing impairment, and a family history of osteoporosis and OI. The hue of sclerae, the whites of the eyes, were assessed. The Beighton scale was used to evaluate joint hypermobility; the hypermobility score ranged from 0 to 9, and generalized joint hypermobility was defined by a cut-off value of 4. For each maneuver, the examiner would first explain and demonstrate before asking the patient to attempt the maneuver (Additional file 1).

The clinical and, when indicated, panoramic radiographic examinations emphasized dental variables associated with OI. Clinically, the following signs were evaluated: retained teeth (failure of eruption), malocclusion, and DGI indicators – pathologic discoloration, attrition, and fractures. Radiographically, these signs were evaluated: number of tooth germs, extended pulp chambers (taurodontism), and DGI indicators – bulbous crowns with cervical constriction, pulpal obliteration, and short roots [5]. Individuals with suspected OI were referred to the national OI multidisciplinary pediatric team at Astrid Lindgren Children’s Hospital for further examination and diagnosis.

The Regional Ethics Committee in Stockholm approved the study (Daybook no. 2014/254–31). All participants and/or their legal guardians signed informed-consent forms. The reporting of this study follows the STROBE checklist for cross-sectional studies.

### Statistical analysis

Prevalence was calculated as number of individuals presenting with DGI/ number of individuals born between 1996 and 2015. Cumulative incidence was calculated as number of new DGI cases reported during 1996−2015/number of newborns at risk of DGI during the time period. Incidence rate (the pace or intensity of accumulation of disease cases) per unit of time was calculated as Number of new cases/number of persons at risk for developing DGI. Data were summarized as proportions, or counts, or means and standard deviations. For categorical variables, the *X*^2^ test determined differences in frequencies of dental aberrations. The independent *t*-test evaluated continuous variables. Two-tailed *p-*values were computed using *p* < 0.05 to denote a significant deviation from the null hypothesis. The results of the examination were structured in a database and statistically evaluated in SPSS (IBM SPSS Statistics 25).

## Results

During the study, pediatric dental clinics reported 45 subjects: 25 females (55%) and 20 males from 28 families. The mean age at the time of the clinical and radiographic evaluations was 7.5 ± 4.4 (range 1.9−19.3) years. Because suspicion of OI was raised in one boy, we excluded him in the calculations of prevalence and incidence, but included him in evaluations of other variables associated with OI.

### Point prevalence, cumulative incidence, and incidence rate of DGI-II

**Fig. 2:**
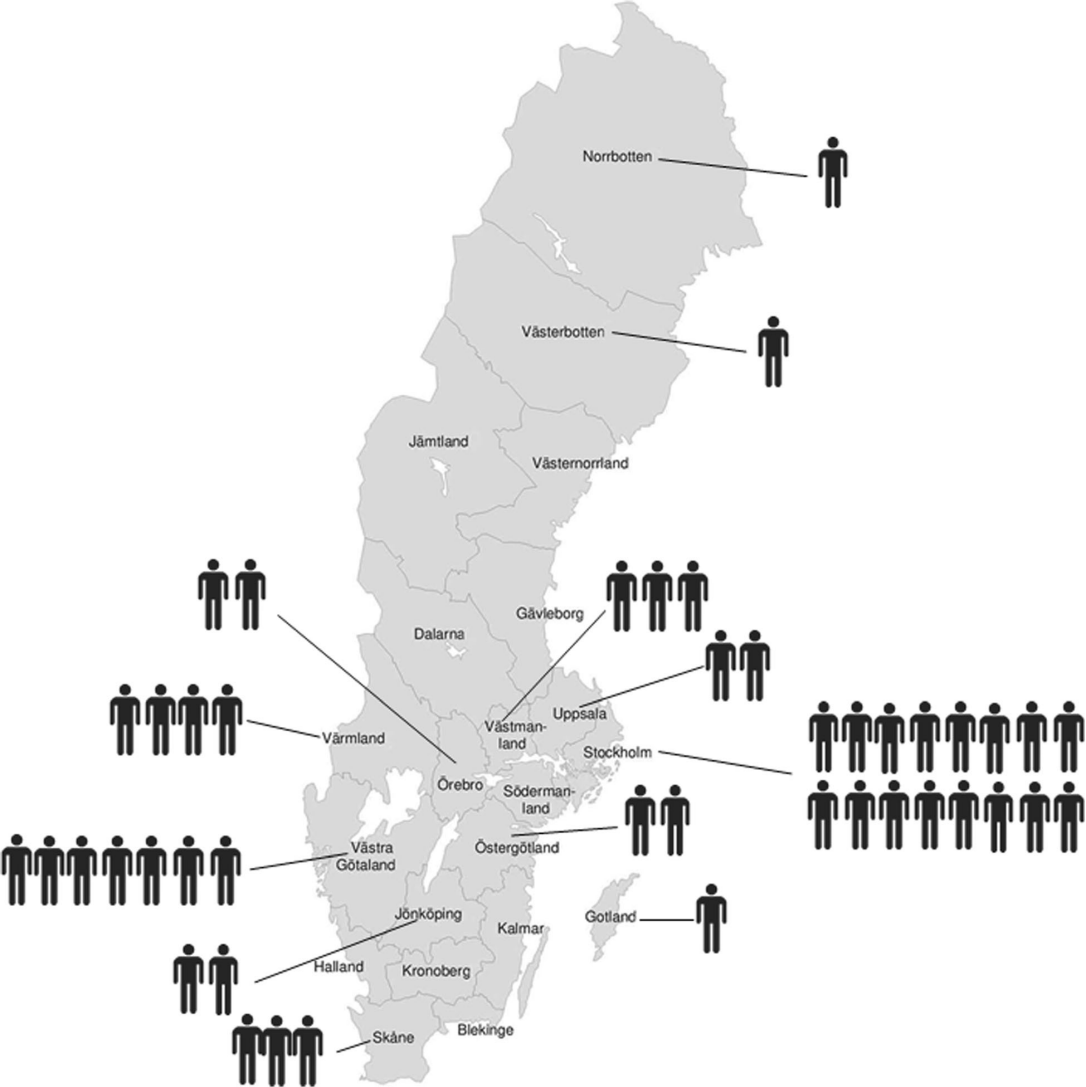
Number of reported children and adolescents with DGI-II (*n* = 44) by county in Sweden

The response rate concerning known cases was 100%; all clinics contacted us. Twelve counties reported cases of resident children diagnosed with DGI-II (Table 1). DGI-II had been diagnosed in 44 children and adolescents (19 males, 25 females) born between 1996 and 2015 (Fig. 2). During this period, there were 2,044,530 births in Sweden [13]. The 2015 point prevalence was thus estimated as 0.0022% (95% CI, 0.0016–0.0029%) (2.2 in 100,000 individuals or 1 in 45,455 individuals). Regional differences occurred. The absolute number of individuals presenting with DGI-II was highest in Stockholm County (*n* = 16), but in relative numbers based on population size, prevalence was highest in Gotland (0.009%) where 10,948 individuals were born during the same period.

**Table 1:** Prevalence of dentinogenesis imperfecta type II (DGI-II) in all Swedish counties (*n* = 21)

County	No. of individuals with DGI-II	No. of individuals born 1996–2015	Prevalence (%)	No. of individuals per 100,000, 95% CI
Norrbotten	1	47,862	0.00209	2.09 (0.23–9.77)
Västerbotten	1	54,668	0.00183	1.83 (0.20–8.55)
Jämtland	0	24,748	0	0 (0–10.2)
Västernorrland	0	47,993	0	0 (0–5.23)
Dalarna	0	54,373	0	0 (0–4.62)
Gävleborg	0	53,872	0	0 (0–4.66)
Värmland	4	50,841	0.00787	7.87 (2.66–18.7)
Örebro	2	62,860	0.00318	3.18 (0.66–10.2)
Västmanland	3	51,668	0.00581	5.81 (1.64–15.5)
Uppsala	2	76,501	0.00261	2.61 (0.54–8.39)
Stockholm	16	493,290	0.00324	3.24 (1.93–5.14)
Södermanland	0	54,186	0	0 (0–4.64)
Västra Götaland	7	346,424	0.00202	2.02 (0.90–3.97)
Östergötland	2	91,357	0.00219	2.19 (0.45–7.02)
Jönköping	2	74,592	0.00268	2.68 (0.56–8.6)
Kalmar	0	43,845	0	0 (0–5.73)
Gotland	1	10,948	0.00913	9.13 (0.99–42.7)
Halland	0	66,827	0	0 (0–3.76)
Kronoberg	0	37,391	0	0 (0–6.72)
Skåne	3	268,070	0.00112	1.12 (0.322–2.99)
Blekinge	0	29,408	0	0 (0–8.54)

The incidence proportion/cumulative incidence of DGI-II was 0.0022% (2.2 per 100,000 newborns per 20 years) in Sweden; the incidence rate was 0.00011% (95% CI, 0.000076–0.00014%) per person-year or 1.1 individuals per 1,000,000 person-years of observation/risk.

### Dental aberrations in children and adolescents with DGI-II

We evaluated 30 individuals clinically according to the examination protocol. The remaining cases (*n* = 14) were diagnosed based on personal communication with the responsible practitioner due to the patient wishing to be excluded from further participation. Panoramic radiographs were available in 63% (19/30) of the examined individuals. Radiographic signs of DGI were confirmed in all 19 cases. The remaining cases were diagnosed based on clinical findings only (*n* = 7); clinical and bite-wing findings (*n* = 2); clinical and histologic findings (*n* = 1); and clinical, radiographic, and histologic findings (*n* = 1). A family history was present in 97% (29/30) of the examined individuals with DGI-II.

Dental agenesis could be evaluated in 23/44 individuals and was present in two individuals (9%). Tooth retention was evaluated in 12/44, present in 2 individuals (17%), pulpal obliteration, evaluated in 24/44 and present in 24 (100%).

### Presence of variables associated with OI in individuals presenting with DGI

Variables associated with OI could be evaluated in the majority of examined individuals, (32/45 regarding bruising and joint hypermobility; 33/45 regarding prolonged bleeding, spraining, fractures, hearing impairment, and blue sclerae). The reasons for missing values were incomplete questionnaire data or lack of cooperation due to low age in individuals evaluated. Eight subjects (25%, 6 boys and 2 girls) stated that they often had bruises and bruised easily. One individual (3%) reported a history of prolonged bleeding. Two individuals (6%) had experience of spraining. One 13-year-old girl reported a previous radius fracture (3%). The fracture was assessed as unassociated with OI as it occurred after falling off a swing. One individual reported hearing impairment (3%) and one examined individual was found to have a scleral hue deviating toward blue or blue-grey tones (3%). Signs of joint hypermobility were found in 50% of the subjects who consented to be examined: 44% (7/16) boys and 56% (9/16) girls. The 30 examined individuals had a mean Beighton hypermobility score of 1.6 ± 2.1 (range 0−6). Two children were too young to cooperate completely. Approximately one quarter (30%; 9/30) of the evaluated individuals had generalized joint hypermobility when a cut-off value of 4 was used. In both sexes, the most commonly affected joints were bilateral involvement of the little finger joint, followed by the elbow, thumb, and knee joints.

### Referral for further medical evaluation of OI

Clinical and radiographic findings in one individual, a 2-year-old boy, gave rise to a suspicion of undiagnosed OI. This boy had a history of bruising easily and was short for his age (− 2 SD). Clinically, he presented with grey-blue sclerae and solitary bruises on his legs. The head circumference of 48 cm, was however, appropriate for his age. Joint hypermobility was present in the right thumb and elbow joint and bilaterally in the knee joints. The deciduous dentition had a grey discoloration, which is typical for DGI. Three deciduous mandibular incisors had been extracted due to root fractures, and histologic evaluation confirmed dysplastic dentin in accordance with DGI. Suspicion of inherited OI was raised due to findings in the mother. The boy was referred to the national OI multidisciplinary pediatric team at Astrid Lindgren Children’s Hospital at Karolinska University Hospital; examination confirmed an OI diagnosis for the boy, and suspected OI in the mother.

## Discussion

We estimated a point prevalence of 0.0022% or 1 in 45,455 individuals for DGI-II in Swedish children and adolescents. The sex distribution of DGI-II was similar and in line with the findings of previous studies [9, 11]. Our finding agrees with predicted effects of the autosomal dominant inheritance pattern seen in DGI-II. One child had several symptoms of OI; further investigation yielded a diagnosis of OI type IV.

Since DGI-II and DGI-III and dentin dysplasia type II are caused by mutations in the same gene and represent phenotypes of differing clinical severity, we used the more recently proposed classification [1–4] rather than the classification proposed by Shields et al. [14] which is based solely on clinical and radiographic signs.

This study reports a significantly lower prevalence of DGI-II than previous reports in the literature. Several factors may explain this difference. The Michigan study by Witkop [9] that reported a prevalence of 0.013−0.017% was done in a region with character of a genetic isolate. Genetic drift (the change in frequency of an allele variant due to random sampling of organisms) might be an important causation factor here. In humans, mutation rates are sometimes great enough to cause substantial drift. Due to the high degree of penetrance of *DSPP* mutations and the autosomal dominant manner in which variants are inherited, the risk of pathogenic variants caused by genetic drift and founder effects (the loss of genetic variation occurring when a population is established from a small number of individuals) is substantially higher in populations with a low degree of migration.

The prevalence we found is also significantly lower than the recent reports of 0.09% [11], 0.057% [10], and 0.1% [12]. The Cassia et al. study [11] used archived radiographs of a cohort of individuals with dental development anomalies. All individuals were patients at the same center. Circumstances were similar in the Yassin et al. study [12], which evaluated children from only one dental clinic. Such approaches are more likely to result in higher prevalences. For comparison, the Malmgren study [15] used a methodology similar to what we used and found a point prevalence of 1 in 82,000 children and adolescents (1.2 per 100,000), which is much nearer to our finding.

We found a family history of DGI-II in 97% of the examined individuals, which agrees with evidence in the literature for a complete penetrance of the disorder and a low degree of de novo mutations [14, 16]. The expression of the disorder in early childhood is substantial, and individuals presenting with a pathogenic variant also exhibit clear signs of the disorder. This enables an early diagnosis in affected children.

The children in our study with DGI-II presented with other dental disturbances. We previously reported a high prevalence of hypodontia (11%) and oligodontia (6%) in children and adolescents with OI [17]. In the present cohort, we observed a hypodontia prevalence of 9%, which is only slightly higher than reported prevalences of 6−8% for the Nordic population in general [18–20]. Another finding in the present study was tooth retention or impaction (third molars excluded) in 17% (2/12). A study by Lagana et al. [21] reported an impaction prevalence of 3.9% in a group of 4706 aged 8−12-years. Retention of permanent second molars is a frequent finding in OI [5, 6, 22], but was not found in any individuals in this cohort. Pulpal obliteration occurred in 100% of the children in the present study. Wide pulp chambers that are later obliterated are frequent findings in DGI and periapical lesions are common compared to unaffected teeth. Pulp necrosis with radiographic signs of periapical disease due to obliteration occurs in only 7−27% of unaffected permanent teeth [23].

OI is a group of rare inherited connective tissue disorders due to abnormal composition or amounts of collagen. Besides bone, other tissues rich in collagen type I may be affected, including the skin, ligaments, and sclerae [24]. The children in the present study presented with variables considered to be potentially important factors for identifying individuals with OI. We observed easy bruising in 25%. Bleeding and easy bruising are common features in heritable connective tissue disorders including OI, Marfan, and Ehlers-Danlos syndromes. The coagulation defect is partly related to the effect of abnormal collagen on platelet–endothelial cell interactions and capillary strength [25–27]. A history of fractures was a rare finding in this study (3%). Fractures is one of the most important factors to evaluate when assessing patients presenting with DGI. A recent study [28] found that fractures were 10.7 times more common in 0−19-year-old children with OI compared to controls. The fracture history of the one patient in our cohort reporting fractures was assessed as compatible with the reported cause of injury.

Individuals with OI type I present with distinctly blue sclerae, which remain blue throughout life. The sclerae of individuals with OI type IV may also be blue at birth and during infancy, but intensity fades within the first years of life [29]. Blue sclerae are also present in many newborns without any associated disorder, and as a finding in infants under 18 months, it is not indicative of OI.

We found varying degrees of joint hypermobility in 50% of the examined children. Joint hypermobility is a common finding in heritable connective tissue disorders. Of individuals with OI, 34−100% are affected depending on the severity of the disorder. Nevertheless, joint hypermobility is also frequently seen in the general population, often decreasing with age [30].

Our examinations found one individual in this cohort who exhibited signs of undiagnosed OI. This boy presented with several of the symptoms associated with OI: easy bruising, growth deficiency, blue-grey sclerae, joint hypermobility (4 points), and DGI. Evaluation of his mother also raised suspicion of OI heredity. The boy was later diagnosed with OI type IV.

During 1996−2015, the dentists in the national OI multidisciplinary pediatric team at Astrid Lindgren Children’s Hospital evaluated 162 individuals with OI. The team found clinical and radiographic signs of DGI-I in 22% (35/162) of the group, indicating that DGI-I is more common than DGI-II (personal communication).

A strength of this study is that our search for cases of DGI-II covered the entire country. Nevertheless, the present study has limitations. The risk of selection bias may have decreased based on our approach to contact clinics that did not report cases. General practicing dentists who discover patients with this type of complex dental developmental disturbances always send referrals to specialist pediatric dental clinics. It is likely that some cases will go undetected, at least for a time. So, we cannot be certain that we were able to discover all cases. Another limitation of the study is the risk of information bias (misclassification) of DGI-II. We consider this risk low as we only included children who had been diagnosed by a pediatric dentist. The phenotype of DGI-II includes pathognomonic features and pediatric dentists are well trained in diagnosis of mineralization disturbances, which may increase the internal validity. The presence of several clinicians involved may though have introduced inter-observer variability for the medical variables being evaluated but the written and illustrated instructions may have decreased this risk.

## Conclusions

In conclusion, this study found a DGI-II prevalence of 0.0022% or 2.2 in 100,000 children and adolescents. This is a lower prevalence than currently has been reported in the literature, but the first study to include cases from all specialist pediatric dental clinics in a country. Among the reported cases of DGI-II, we identified one child with undiagnosed OI. This underscores the importance of evaluating OI symptoms in children and adolescents with DGI-II.

## Additional file

Additional file 1: Examination protocol Prevalence study of dentinogenesis imperfecta (DGI). (DOCX 250 kb)

## Abbreviations

DGI: Dentinogenesis imperfecta
DGI-I: Dentinogenesis imperfecta type I
DGI-II: Dentinogenesis imperfecta type II
DSPP: Dentin sialophosphoprotein
OI: Osteogenesis imperfecta
