# Supplementary material for: Dentinogenesis imperfecta type II in Swedish children and adolescents
Source: Orphanet J Rare Dis. 2018 Aug 22;13:145. doi: 10.1186/s13023-018-0887-2 (PMC6106925; doi:10.1186/s13023-018-0887-2)
Supplement: Supplementary file 1 — Examination protocol Prevalence study of dentinogenesis imperfecta (DGI). (DOCX 250 kb) [file 13023_2018_887_MOESM1_ESM.docx]

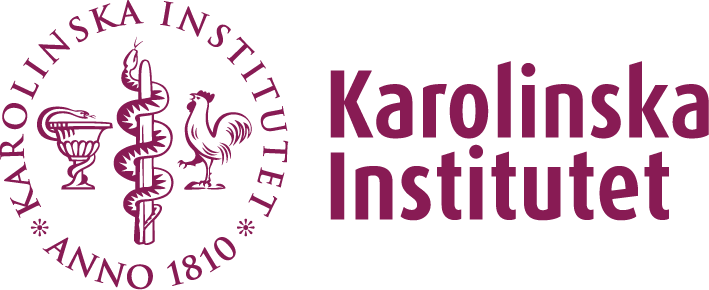


Additional file 1

**Examination protocol**

**Prevalence study of dentinogenesis imperfecta (DGI)**

**Patient name and personal identity number:**

**Address:**

**Phone number:**

**Consent obtained from** Guardian Patient

**Date of examination:**

**QUESTIONS FOR THE PATIENT OR THE GUARDIAN**

**Do you (does your child) bruise easily?** Yes No

**Do you experience that you have (your child has) prolonged bleeding?** Yes No

**Do you (does your child) sprain easily?** Yes No

**Have you (has your child) had any bone fractures?** Yes No

**If you have (your child has) had a bone fracture, how many times?**

……………..times.

**What bones have you (has your child) broken and how?**

……………………………………………………………………………………

**Do you (does your child) have a hearing impairment?** Yes No

**Do you have a family history of osteoporosis?** Yes No

**Does anyone in the family have osteogenesis imperfecta (OI)?**

Yes No

**If yes, what is their name and how are you related?.........………………....**

**………………………………………………………………………………………**

**Does anyone in the family have brittle teeth (DGI)?**

Yes No

**Name of the treating physician/medical center/hospital:**

**CLINICAL ASSESSMENTS**

**Does the patient exhibit joint hypermobility?**  Yes No

**If yes, which joints are affected? Specify whether on the right or left side**

**……………………………………………………………………………………………………………………………………………………………………………...**

**Does the patient have characteristic changes in the color of the whites of the eyes (the sclerae)?** Yes No

**How tall is the patient? …………………**

**Does the patient have tooth agenesis?**

Yes No

**If yes, which teeth are missing? …………………**

**Does the patient have retained second molars?**  Yes No

**Does the patient have other retained teeth?**  Yes No

**If yes, which teeth are retained? …………………**

**Does the patient have unusually large pulp chambers?**  Yes No

**Does the patient have obliterated pulps?**  Yes No

**Does the patient exhibit any malocclusion?**  Yes No

**If yes, what type?**

**……………………..……………………………………………………………….**

**Does the patient exhibit any other dental anomalies?** Yes No

**If yes, which ones?**

**…….………………………………………………………………………………..**

**………………………………………………………………………………………**

**Examining dentist (name, clinic, and phone number):**

**Assessment of the sclerae**

Patients with osteogenesis imperfecta sometimes exhibit a discoloration of the whites of the eyes (sclerae). The discoloration is often blue and is caused by an altered translucency due to the collagen defect. It is thus important to assess any tinge of discoloration of the sclera. Please record your findings in the examination protocol.


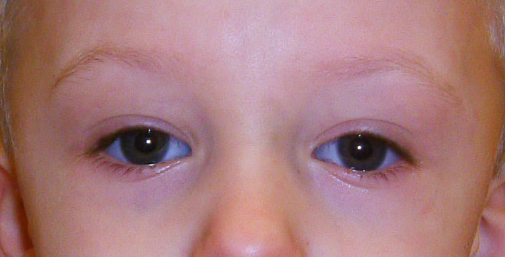


**Assessment of joint hypermobility**

This assessment is based on the Beighton scale. Please note which joints are affected. Nine points are possible. A detailed description of the maneuvers is shown below.

1) Passive dorsiflexion of the little fingers beyond 90 degrees (1 point per little finger)

2) Passive apposition of the thumbs to the flexor aspects of the forearms (1 point per thumb)

3) Hyperextension of the elbows more than 10 degrees (1 point per elbow)

4) Hyperextension of the knees more than 10 degrees (1 point per knee)

5) Forward flexion of the trunk, with knees straight, so that the palms of the hands rest easily on the floor (1 point)

**
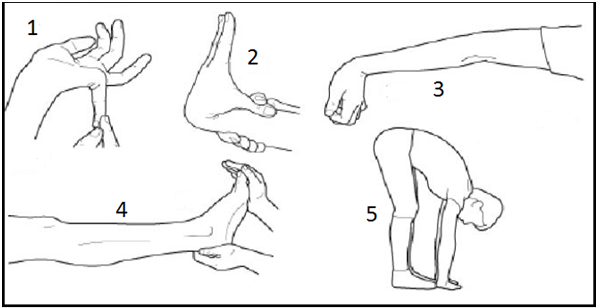
**
